# Supplementary material for: MetaRibo-Seq measures translation in microbiomes
Source: Nat Commun. 2020 Jun 29;11:3268. doi: 10.1038/s41467-020-17081-z (PMC7324362; doi:10.1038/s41467-020-17081-z)
Supplement: Supplementary file 10 — Supplementary Data 7 [file 41467_2020_17081_MOESM10_ESM.zip › File2/Confidence_High_Taxonomy/142295_out.krona.html]

Javascript must be enabled to view this page.

members
magnitude
magnitudeUnassigned
count
unassigned
taxon
rank

142295\_out

2

superkingdom
2
2

2
1239
phylum

class
2
186801

186802
2
order

species
1
1897045

SRS143523\_contig\_number\_contig-100\_5.159649

family
186803
1

species
1

SRS045826\_contig\_number\_19635
658087
